# A Simple Denture-Marking Technique for Patients Residing at Old Age Homes

**DOI:** 10.7759/cureus.30367

**Published:** 2022-10-16

**Authors:** Shreya Colvenkar, Aditya Mohan Alwala, Ramesh Kunusoth, Shalini Sampreethi, Shravya Devera Shetty

**Affiliations:** 1 Department of Prosthodontics, MNR Dental College and Hospital, Sangareddy, IND; 2 Department of Oral and Maxillofacial Surgery, MNR Dental College and Hospital, Sangareddy, IND; 3 Department of Public Health Dentistry, MNR Dental College and Hospital, Sangareddy, IND

**Keywords:** prosthodontics, labeling dentures, forensic, geriatric, marker, photograph, denture

## Abstract

Denture marking helps to identify unknown individuals in social and forensic scenarios. This article describes a simple technique of incorporating a patient's photograph with details in the patient’s denture. The photographic marker will help in quick identification in old age homes as it is easily readable by a layperson, playing an important role in day-to-day identification. This technique can be used to label both new and existing dentures that are not marked. The simple technique will ensure positive identification of denture wearers.

## Introduction

Denture marking plays a vital role in the identification of the living as well as the deceased. Former is important to identify individuals suffering from amnesia and unconsciousness. The latter is important for the identification of recovered bodies of mass disasters such as tsunamis, earthquakes and aviation crashes. Denture marking plays a crucial role in old age homes where staff can misplace, mix, and even lose dentures, especially during the cleaning of prostheses [1,2]. Providing a replacement denture for geriatric patients is a continuous struggle as they have to adjust to the new fit of dentures. The greatest difficulty is for elderly patients who are physically frail and suffering from systemic diseases such as Alzheimer’s and Parkinson’s disease. It also requires the time and will to adapt to new learning sequences. Considering all these factors, it is important to mark dentures in old-age homes.

Various methods of labelling dentures have been summarized in the literature, which can be divided into surface marking [3,4] and inclusion techniques [5-9]. Surface marking techniques include marking dentures with pen or engraving. In surface inclusion, a tiny code, metallic or non-metallic, with patient details is embedded in the denture. These methods vary depending on the method of fabrication as well as readability.

This article describes a simple technique of incorporating a patient's photograph in dentures. The photographic marker will help in the quick identification of dentures in old age homes and hospital setups.

## Case presentation

A 65-year-old illiterate male patient reported to the department of prosthodontics for the replacement of his lost complete maxillary and mandibular denture. History revealed the patient was suffering from Alzheimer’s disease and couldn’t remember where it was misplaced. The patient's straightforward request was to insert some identification mark in the denture so that it could be easily retrieved if lost or misplaced. Various types of denture-marking systems were discussed with the patient as well as the patient’s caregiver. The patient requested a photographic marker, which is easily readable.

Technique

**Figure 1:**
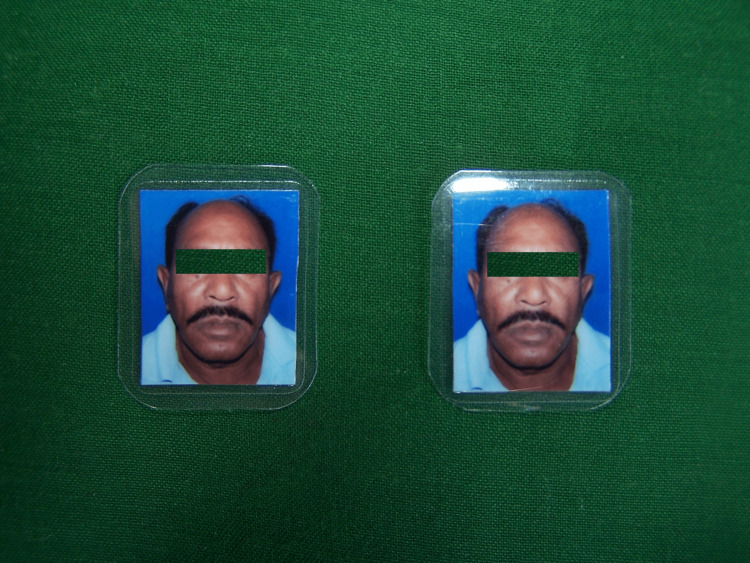
Laminated photograph of the patient

The patient's photograph was printed on a glossy photo paper of size 10x10mm. On the opposite side of the photograph, the patient's name, age, and Aadhar number were added. Aadhaar is a 12-digit individual identification number issued by the Unique Identification Authority of India on behalf of the Government of India to all residents. The photograph was laminated with lamination sheet of 180 microns (Polycrom Korean Sheet, Polycrom, Bangalore, India) to prevent smudging of the photograph (Figure 1).

**Figure 2:**
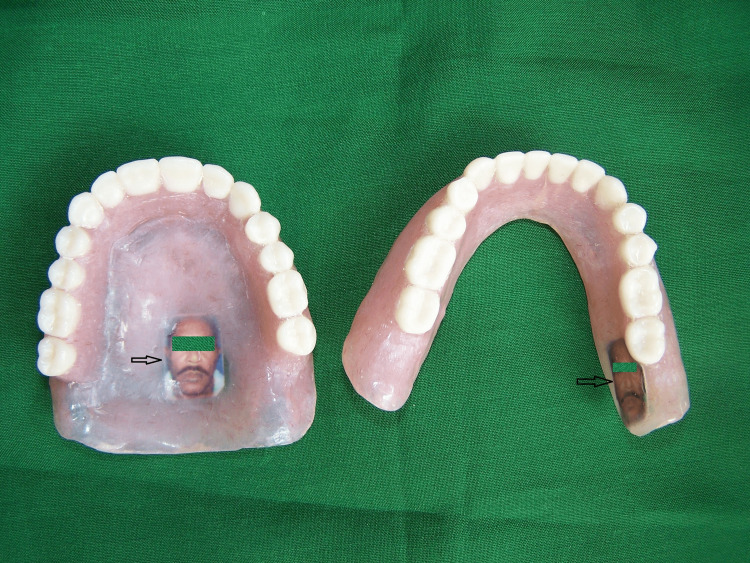
Denture with patient photograph

The try-in stage was finished and the laboratory procedures for denture fabrication till the dewaxing stage were completed. The photographic marker was inserted in the denture during the packing of the heat-cure acrylic resin, such that the photograph faced the polished surface of the denture (Figure 2).

**Figure 3:**
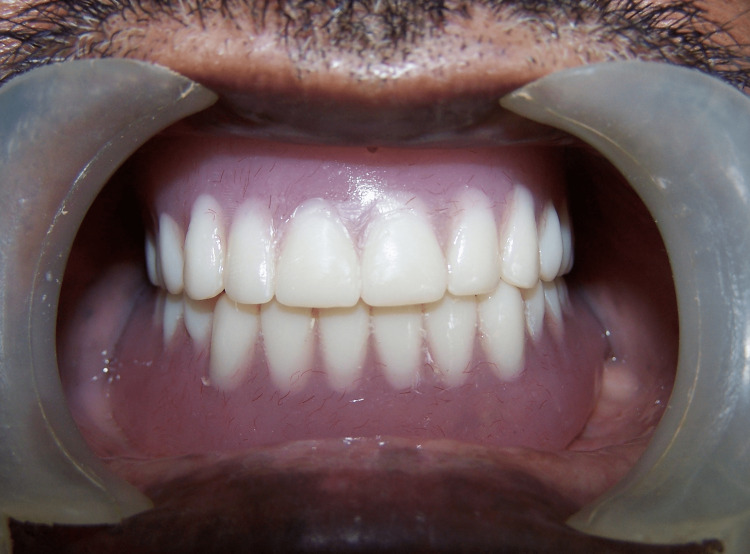
Intraoral view of patient with dentures

After trial packing, a small area of heat cure acrylic resin was cut corresponding to the size of the marker in the distolingual flange of the mandibular denture and the posterior region of the palate of the maxillary denture. The marker was carefully sandwiched between two layers of clear heat-cure clear acrylic resin dough. This was followed by heat curing of the denture using a conventional technique. The dentures were finished and polished and delivered to the patient (Figure 3).

## Discussion

The marking of dentures is vital to confirm the positive identification of a denture wearer. In forensic scenarios, dentures at the scene provide vital clues in recognizing the denture wearer. Various methods of marking dentures have been mentioned in the literature with advantages as well as disadvantages [3-9]. Surface marking is simple and economical but not durable [3,4]. In the surface inclusion technique, metallic or non-metallic makers are inserted in the prosthesis [5]. Microchips [6], microSD cards [7], and quick response (QR) code [9] identification can store huge amounts of information but require a hand-held reader or mobile to read the information. The lenticular method is technique sensitive, and information can never be changed [8]. 

When patients misplace or lose their dentures, a tiny identification mark will help in the quick return of the denture to the concerned individual. Dentures need to be returned immediately to restore function and esthetics as they can have an undermining effect on the quality of life [10]. Moreover, elderly patients may have difficulty adapting to new prostheses. In India, 20% of elderly people live alone in old age homes and the remaining 81% live with their families [11]. But with urbanization, and the breakdown of the traditional joint family system, caring for the aged will become institutionalized [12]. This will warrant the routine use of denture markers. Hence, it is essential that markers are easily readable.

The use of photographs in contrast to the smaller-sized alpha-numeric characters will ensure quick day-to-day identification. It also prevents the exchange of dentures among illiterate and multi-lingual denture wearers in old-age facilities. Also, photographic markers with patient information recovered from the deceased, when shown to the kin, may ensure quick identification.

Lamination was done with high-quality transparent polyester sheets. This prevented the ink of the photograph from blotting on contact with the methyl methacrylate. There was no wrappage or shrinkage of the laminated sheet and photograph during denture processing.

The small size of the marker did not hinder the aesthetics, oral function, and strength of the denture. This technique can be used to label both new and existing dentures that are not marked. The marker can be inserted in existing dentures on the buccal flange of the maxillary denture and lingual flange of the mandibular denture which are esthetically acceptable. The marker can be inserted by making a small depression with carbide bur, corresponding to the size of the marker. The marker is inserted and sealed with clear auto-polymerized acrylic resin. The only disadvantage is it's not resistant to high temperatures. The simple technique will ensure positive identification of denture wearers.

## Conclusions

Denture marking plays a crucial role in positive identification of denture wearers either living or dead. This article describes a simple and quick technique of incorporating a photograph in the patient’s denture. The photographic marker is easily readable by a layperson, playing an important role in day-to-day identification in old age homes. This technique can be used to label both new and existing dentures that are not marked. 
